# Harlequin color change in preterm infant

**DOI:** 10.11604/pamj.2015.22.119.8056

**Published:** 2015-10-12

**Authors:** Emira Ben Hamida, Imene Dahmane Ayadi

**Affiliations:** 1 Department of Neonatology, Charles Nicole Hospital, Tunis El Manar University, Tunis, Tunisia

**Keywords:** Harlequin color change, dermatology, neonate, preterm

## Image in medicine

Harlequin phenomenon is a rare event that consists of sudden and transient episodes of demarcated erythema forming on the dependent half of the body of newborns, with pallor of the uppermost half. The infant appears as if a line was drawn on the midline dividing two hemibodies. It occurs preferentially in lateral position. It is most common in term newborns, rare cases were reported in preterm. The etiology is unknown; immaturity of the sympathetic peripheral vascular tonus was evoked as a possible cause. The diagnosis is clinical; no treatment is needed. The recognition of the benign condition is required to avoid unnecessary investigations. We report a female newborn, the second of a twin pregnancy, born premature at 34 weeks of gestation by caesarean section. Apgar's scores were 9 and 10 at 1 and 5 minutes, respectively. Body weight was 1.640 kg (11h percentile); the neonatal examination was normal. Initially the neonate was admitted to neonatal intensive care unit for nutritional support. On 5th day of life she developed respiratory distress related to nosocomial sepsis leading to intubation and mechanical ventilation. She didn't develop hemodynamic instability. On 16th day of life, she developed suddenly unilateral erythema with simultaneous contralateral pallor with a clear demarcation line entirely separating left and right sides of the body. The infant was in supine position. This erythema resolved spontaneously within three minutes. Newborn's monitoring revealed no concomitant changes in blood pressure, respiratory or heart rate. We noted only one episode.

**Figure 1:**
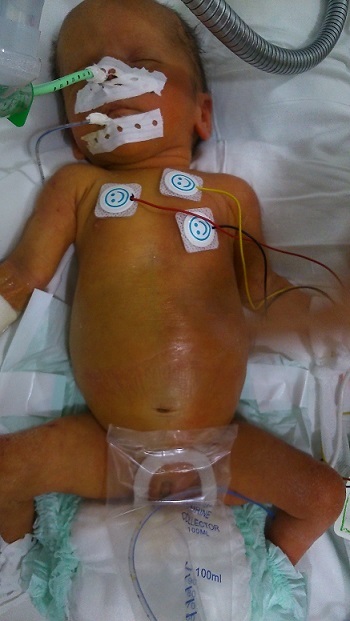
Harlequin color change

